# Ad26.COV2.S Vaccine-Induced Thrombocytopenia Leading to Dural Sinus Thrombosis and Intracranial Hemorrhage Requiring Hemicraniectomy: A Case Report and Systematic Review

**DOI:** 10.7759/cureus.28083

**Published:** 2022-08-16

**Authors:** Samuel R Daly, Anthony V Nguyen, Jose M Soto, Awais Z Vance

**Affiliations:** 1 Neurological Surgery, Baylor Scott & White Medical Center-Temple, Temple, USA

**Keywords:** intracranial hemorrhage, neurosurgical decompression, craniectomy, cerebral venous sinus thrombosis (cvst), vaccine-induced thrombosis and thrombocytopenia (vitt), covid-19, ad26-cov2-s

## Abstract

The coronavirus disease 2019 (COVID-19) pandemic has claimed nearly 5.5 million lives worldwide. Adenovirus-based vaccines are safe and effective, but they are rarely associated with vaccine-induced thrombosis and thrombocytopenia (VITT) as well as cerebral venous sinus thrombosis (CVST). We conducted a systematic literature search of intracerebral hemorrhage (ICH) secondary to CVST associated with VITT from the Ad26.COV2.S vaccine, and we present the first case of this pathology in the reviewed literature of a patient who required neurosurgical decompression. The systematic literature review was completed on December 19, 2021, by searching PubMed and Ovid for articles with primary data on CVST associated with VITT following the Ad26.COV2.S vaccine. We also specifically searched for cases that required neurosurgical intervention. Articles were independently screened by two authors, and both secondary and tertiary searches were done as well. Descriptive statistics were collected and presented in table form. Nine studies were identified that met inclusion criteria. There were no cases identified of patients who underwent neurosurgical decompression after developing this pathology. We thus present the first case in the reviewed literature of a patient who developed ICH after receiving the Ad26.COV2.S vaccine and underwent decompressive hemicraniectomy. Despite severe thrombocytopenia and prolonged intensive care, the patient was discharged to neurorehabilitation. There is a much greater risk of CVST and ICH during COVID-19 infections than from the vaccines. However, as booster vaccines are approved and widely distributed, it is critical to make prompt, accurate diagnoses of this vaccine-related complication and consider neurosurgical decompression.

## Introduction

The coronavirus disease 2019 (COVID-19) pandemic has been responsible for more than five million deaths globally as of December 2021, approximately 800,000 of which have occurred in the United States [1]. The relative infectivity and mortality of COVID-19 have driven research and production of various efficacious vaccines, which utilize either messenger RNA (mRNA) or adenoviral vectors [2].

Adenoviral vector vaccines have been shown to be safe through rigorous clinical trials, but they are associated with rare serious complications [3]. One potential complication that has been cited is the development of vaccine-induced thrombosis and thrombocytopenia (VITT) [4]. VITT frequently involves the cerebral veins or dural sinuses, which can lead to venous hypertension and intracerebral hemorrhage (ICH), resulting in approximately 50% mortality [4,5]. Although the mechanism underlying the development of VITT has not been fully elucidated, one potential hypothesis involves the migration of an adenovirus vector into the bloodstream with subsequent activation of platelets, release of platelet factor-4 (PF-4), and production of anti-PF-4 antibodies [6].

Here, we present a case of intraparenchymal hemorrhage (IPH) secondary to cerebral venous sinus thrombosis (CVST) associated with VITT from the Ad26.COV2.S vaccine that required neurosurgical decompression and hematoma evacuation. We also present a systematic review of the literature on CVST from VITT associated with the only adenoviral vector vaccine in emergency use authorization in the United States (Ad26.COV2.S) [7], in order to determine if neurosurgical decompression has been reported as a viable option for patients suffering significant intracerebral hemorrhage from this pathology.

## Case presentation

**Figure 1:**
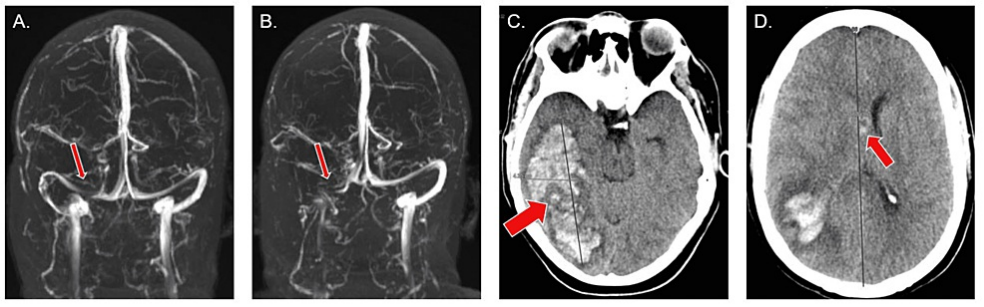
Selected relevant neuroimaging. (A) MRV on hospital day four, revealing a non-obstructing thrombus at the junction of the right transverse and sigmoid sinuses (see arrow). (B) MRV on hospital day 10 with interval progression of the thrombus in the right transverse and sigmoid sinuses without flow (see arrow). (C) CT without contrast, showing a large occipital and temporal lobe intraparenchymal hematoma. (D) The different axial cut of the same CT without contrast shows 7 mm of midline shift (arrow) and intraventricular extension of the hematoma (arrow). MRV: magnetic resonance venogram; CT: computed tomography; mm: millimeters.

A 44-year-old female with a history notable for hypertension and tobacco use had received the Ad26.COV2.S vaccine eight days prior presented to the emergency department (ED) of an outside hospital with an acute onset of abdominal pain, nausea, and vomiting. Abdominal imaging was negative at that time, and she was discharged home. Lab values are listed in Table 1. She returned to the same ED the following day for worsening abdominal pain, and a new work-up demonstrated bilateral adrenal hemorrhage and thrombocytopenia (platelet count of 87 × 10^9^/L). She was admitted, and the department of hematology was consulted, because she met the clinical criteria for VITT [8]. PF-4 antibodies were positive, and she was transferred to a higher level of care at a second outside hospital. When she arrived to the second hospital, she was given intravenous immunoglobulin (IVIG), and a magnetic resonance venogram (MRV) of her head demonstrated a non-occlusive right transverse and sigmoid sinus thrombosis (Figure 1A). She was started on argatroban and transitioned to fondaparinux four days later. After two days on fondaparinux, she developed a headache, so a repeat MRV of her head was done. It revealed interval enlargement of the thrombus with complete occlusion of the sigmoid sinus (Figure 1B). A CT of her head was negative at that time. She was transitioned back to argatroban and transferred to our institution for neurosurgical consultation.

**Table 1:** Laboratory values from significant time points during hospitalization. Lab results from the patient at initial presentation to the emergency department, with peak values (*trough values for the platelet count), and lab results on the day of the craniectomy. D#: day after initial presentation; mcg: micrograms; mL: milliliter; mg: milligram; dL: deciliter; L: liter; INR: internal normalized ratio; aPTT: activated partial thromboplastin time; s: seconds; PCR: polymerase chain reaction; PF-4: platelet factor-4.

	Initial presentation	Peak value	Day of craniectomy (D15)	Reference range
D-dimer	1.94	>20.00 (D4)	6.81	0-0.5 mcg/mL
Fibrinogen	488 (D4)	561 (D19)	306	200-400 mg/dL
Platelet count	151	5 (D19)*	43*	150-400 × 10^9^/L
INR	1.1 (D3)	2.1 (D13)	1.0	0.9-1.1
aPTT	22.3	109.4 (D5)	29.2 s	23.0-36.0 s
COVID-19 test	Negative (PCR)			
PF-4 antibodies	Positive: 2.138 (HD3)			0-0.399

She arrived at our institution 18 days following the administration of the Ad26.COV2.S vaccine and 10 days following the onset of symptoms. Upon arrival, her platelet count was 46 × 10^9^/L and magnetic resonance imaging (MRI) of her head demonstrated edema and meningeal enhancement of the right posterior temporal lobe, suggestive of venous hypertension. On hospital day four, her mental status deteriorated, and she developed non-reactive anisocoric pupils. She was intubated and started on hypertonic fluids. A repeat CT of her head demonstrated a large right occipitotemporal IPH with approximately 7 mm of midline shift (Figure 1C, 1D). Her platelet count was 43 × 10^9^/L. Argatroban was paused, and she was taken emergently to the operating room for a decompressive hemicraniectomy and hematoma evacuation.

On the post-operative day (POD) 1, an external ventriculostomy drain (EVD) was placed due to persistent intracranial hypertension. On POD 2, her argatroban was restarted after a head CT demonstrated the stability of the evacuated hematoma. On POD 3, her platelet count was 6 × 10^9^/L despite multiple platelet transfusions, so IVIG was administered. The patient's platelet count did not respond, and due to concern about inducing hypercoagulability with continued transfusions, plasma exchange was started on POD 4. Her platelet count improved to 57 × 10^9^/L, and daily plasma exchange was continued. On POD 5, paralytics-and subsequently phenobarbital-induced burst suppression-were required to manage her refractory intracranial hypertension. Phenobarbital was weaned over four days, and paralytics were weaned the following day. Her EVD was eventually replaced with a lumboperitoneal shunt due to persistently high output from the drain. The patient was admitted to the ICU for a total of 83 days, and in addition to daily critical care, she required intermittent treatment with argatroban, frequent plasmapheresis, and weekly rituximab. Although her rehabilitation is still in progress, the patient is able to interact with other people, demonstrate purposeful movements, and follow commands.

## Discussion

Literature review: methods

**Figure 2:**
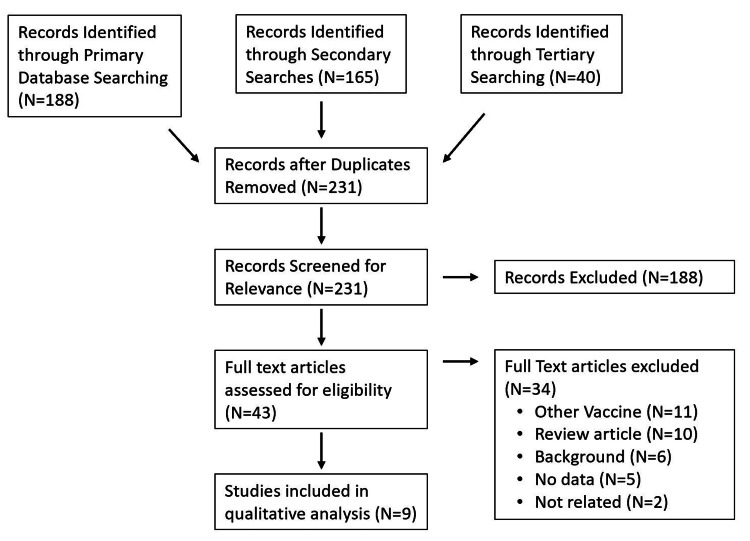
Flowchart of the review methodology. After accounting for duplicates, a total of 231 articles were screened for inclusion. Of those, nine studies were ultimately included in the qualitative analysis. N: number of articles.

A systematic literature search was completed utilizing both the PubMed and Ovid databases. The searches were done on December 19, 2021, using the following broad search terms: ((SARS-COV) OR ("SARS COV") OR ("novel coronavirus") OR (nCoV) OR ("2019-nCoV") OR (COVID) OR (SARS-CoV-2) OR (COVID-19)) AND (“vaccine”) AND ((“cerebral venous sinus thrombosis”) OR (“Sinus thrombosis”) OR (“dural sinus thrombosis”) OR (“venous sinus thrombosis”)). Articles were included if they had primary data on CVST associated with VITT following the administration of the Ad26.COV2.S vaccine. Review articles were excluded. All articles were independently screened at each step for inclusion by two authors (SD and AN; Figure 2). Discrepancies were addressed via discussion with a third author (JS). Secondary and tertiary searches were also completed by screening the citations from the articles that were marked for inclusion by at least one author after a full-text review (Figure 2). Descriptive data were collected and summarized from each article, and the quality of evidence for each included article was rated by a single author (SD) utilizing the rating scheme modified from the Oxford Centre for Evidence-based Medicine.

A second systematic literature search was completed utilizing both the PubMed and Ovid databases for articles on CVST associated with VITT following the administration of the Ad26.COV2.S vaccine that required neurosurgical decompression. The search was done on December 19, 2021, by adding the following terms to the above search: “AND ((“craniotomy”) OR (“craniectomy”) OR (“hemicraniectomy”)).” The review process for this search was identical to the first one.

Literature review: results

CVST Associated With the Ad26.COV2.S Vaccine 

The primary literature search resulted in 95 articles after duplicates were removed, which were screened independently by two authors (SD and AN). This primary review produced eleven articles that were marked for inclusion by at least one reviewer, so the secondary search included 165 citations of these eleven articles. The secondary search resulted in three additional articles that were marked for inclusion by at least one reviewer, so a tertiary search with the 44 citations of those articles was also done, which did not reveal any further relevant articles. After discussion with a third author (JS) about discrepancies, a total of nine articles were found to meet the study inclusion criteria. About 40 patients were included in these nine articles, although an exact number is difficult to accurately establish due to the potential dual reporting of cases. All nine articles had a level of evidence rating of 4 or 5.

The first reported case of CVST associated with the Ad26.COV2.S vaccine occurred during the phase III study for the vaccine [9]. This case was in a 25-year-old male who had a seizure 19 days after receiving the vaccine [10], and he was found to have a transverse sinus thrombosis associated with cerebral hemorrhage two days later. He tested positive for anti-PF-4 antibodies. The phase III study was paused at that time, and the case was reviewed. However, it was determined that his CVST was due to multiple pre-disposing factors rather than the vaccine, and the study was continued.

Following FDA approval of the vaccine, there were case reports of CVST associated with VITT [11,12], which were later included in a case series published in JAMA [13]. This case series included 12 patients for whom CVST and thrombocytopenia were reported as vaccine reactions to the Vaccine Adverse Event Reporting System (VAERS) in the United States. All patients were white women, from 18 to 60 years old, who started experiencing a headache 6-15 days after receiving the vaccine. Seven of the 12 had a risk factor for hypercoagulability. None of the 12 patients had previous exposure to heparin, and all 11 patients that were tested for the PF-4 antibody were positive. Six patients were initially treated with heparin and subsequently switched to non-heparin-based anticoagulation, two were treated with argatroban, two were treated with bivalirudin [11,12], and seven patients also required IVIG. Seven of the patients had an intracerebral hemorrhage, but none underwent neurosurgical intervention. At the time the study was published, two additional patients had been identified via the VAERS that had CVST potentially related to VITT, one of which was presumably written up in a case report that month [14]. This patient met the criteria for VITT and was diagnosed with CVST, but she had not required surgery at the time of publication.

There have also been cases of CVST associated with VITT from the Ad26.COV2.S vaccine reported outside the US. The first published case identified in our search outside the US was of a 37-year-old female with no pre-existing risk factors for thrombosis who had been started on therapeutic low-molecular-weight heparin 10 days after the vaccine for a popliteal vein thrombosis [15]. She presented two days later with multiple bilateral IPHs, a platelet count of 50 × 10^9^/L, low fibrinogen, and elevated d-dimer. A subsequent MRI revealed a superior sagittal sinus thrombosis and worsening intracranial hemorrhages. Despite the placement of an EVD and aggressive ICU care, she deteriorated neurologically and was declared brain dead the following day. A second case was included in a multinational cohort study of patients with CVST associated with thrombocytopenia within 28 days of receiving one of the COVID-19 vaccines [16]. This patient was positive for PF-4 antibodies and had a positive platelet activation assay, but data on the specific treatment regimen and outcome of this patient was not reported, as it was aggregated with patients that had received the other three vaccines. There are also two articles published with data from the EudraVigilance database in Europe that include patients who suffered a CVST and had thrombocytopenia (defined as platelet count <150 × 10^9^/L) within 28 days of receiving one of the COVID-19 vaccines. The first includes 23 patients that met these criteria after receiving the Ad26.COV2.S vaccine [17]. They report a mortality rate of 17% (4/23), but further data from these cases are not available, as the remainder of them are aggregated with cases of patients who had received one of the other COVID-19 vaccines. The second of these publications reported the absolute risk of CVST with thrombocytopenia after the Ad26.COV2.S vaccine as 0.7 per million first doses (95% CI 0.2-2.4) [18]. However, only two cases were included in this analysis, so the absolute risk may be slightly underestimated.

Neurosurgical Intervention for CVST Associated With the Ad26.COV2.S Vaccine

The literature search resulted in two articles after duplicates were removed. Neither article included cases that met the inclusion criteria of CVST associated with VITT from the Ad26.COV2.S vaccine requiring neurosurgical decompression.

However, the two excluded studies from this search are worth mentioning as they describe cases of CVST after another adenovirus vaccine-ChAdOx1-and both required decompressive craniotomies [19,20]. Both cases are of young patients (27-year-old male and 32-year-old female) who presented with headaches to the ED within two weeks of receiving the ChAdOx1 vaccine. One patient was found to have a right transverse sinus thrombosis, and he was treated with dabigatran and IVIG. He subsequently developed an IPH and required a decompressive craniotomy as well as an EVD to control his intracranial pressure. Despite those measures, he continued to deteriorate, and medical management was withdrawn. The second patient was found to have a large right parietal lobe hemorrhage associated with thromboses of her left transverse sinus, sigmoid sinus, and partial sagittal sinus thrombosis. She was treated with enoxaparin and hypertonic solutions, but she deteriorated and underwent emergent hemicraniectomy. She became thrombocytopenic following surgery, which improved with IVIG, and she was eventually discharged to neurorehabilitation (Table 2).

**Table 2:** Studies meeting inclusion criteria for review. Summary of studies reporting on patients with CVST associated with VITT after Ad26.COV2.S vaccine. Of note, none of the patients were treated with neurosurgical decompression. N: number of patients with CVST associated with VITT after receiving the Ad26.COV2.S vaccine; PMH: past medical history; ICH: intracerebral hemorrhage; PF-4 Ab: platelet factor 4 antibody; N/A: not available. *Includes patients from Yahyavi-Firouz-Abadi et al. and Clark et al. **Eleven of 12 were tested for PF-4 Ab, and all 11 were positive.

Study	Type of study, sample size	Relevant patient characteristics	PF-4 Ab	Clinical management	Outcome	Level of evidence
Sadoff et al. [9]	Phase III study for the vaccine, N=1	Twenty-five-year-old male with no PMH, who was diagnosed with a CVST and associated ICH 21 days post-vaccination.	(+)	Thrombectomy and stent placement, followed by thrombectomy and venoplasty due to the second CVST.	Not reported	5
Yahyavi-Firouz-Abadi, et al. [11]	Case report, N=1	Thirty-year-old female, who was diagnosed with a CVST 15 days post-vaccination.	(+)	Argatroban and bivalirudin, and the patient was discharged on apixaban.	Discharged home	5
Clark et al. [12]	Case report, N=1	Forty-year-old female with no PMH, who was diagnosed with a CVST 12 days post-vaccination.	(+)	Bivalirudin, IVIG, and prednisone.	Resolution of symptoms	5
See et al. [13]	Case series, N=12	Eighteen to sixty-year-old females,* of which seven had hypercoagulability risk factors, who were diagnosed with a CVST. (Seven had associated ICH.)	(+)**	Variable medical management with anticoagulation, including bivalirudin, argatroban, and non-heparin-based anticoagulation. (Seven received IVIG.)	Death (3), discharged home (4), remained hospitalized (5)	4
Muir et al. [14]	Case report, N=1	Forty-eight-year-old female with no PMH, who was diagnosed with a CVST and associated ICH ~14 days post-vaccination.	(+)	Unfractionated heparin was switched to argatroban. Also received IVIG.	Remained critically ill at the time of publication	5
Rodriguez et al. [15]	Case report, N=1	Thirty-seven-year-old female with no PMH, who was diagnosed with a CVST 12 days post-vaccination.	(+)	Low-molecular-weight heparin was switched to danaparoid. Also received IVIG and dexamethasone, and required EVD placement.	Brain death	5
Sanchez van Kammen et al. [16]	Cohort study, N=1	(+) Platelet response assay	(+)	Not reported independently of data from other vaccines.	Not reported. independently of data from other vaccines.	5
Van de Munckhof et al. [17]	Cohort study, N=23	Not reported independently of data from other vaccines.	N/A	Not reported	Mortality: 17% (4/23)	4
Krzywicka et al. [18]	Population study of overall risk, N=2/3,023,204 vaccines	The only data reported was the absolute risk of CVST with thrombocytopenia after Ad26.COV2.S vaccine: 0.7 per million first doses (95% CI 0.2-2.4).	N/A	Not reported	Not reported	4

Discussion

We have presented the first case in the reviewed literature of neurosurgical decompression for IPH secondary to CVST from VITT associated with the Ad26.COV2.S vaccine. While the patient's medical course was complex and prolonged, she has been discharged from the hospital to neurorehabilitation in stable condition with gradually improving neurologic function. Of note, both patients discussed with this pathophysiology following the other adenovirus vaccine (ChAdOx1) had higher platelet counts at the time of decompressive craniotomy than our patient (43 × 10^9^/L), and the patient that survived had a platelet count >100 × 10^9^/L [21]. This case demonstrates that decompressive craniectomy is a viable, potentially lifesaving procedure in this patient population.

The results of our literature search are severely limited by the quality of evidence, which is a direct result of the extremely low number of patients that suffer from this vaccine-related complication. Our literature search may also be slightly limited by the number of databases used. However, all COVID-19-related articles are currently PubMed indexed, and our secondary and tertiary searches included many sources that were outside of the databases originally included. We were also able to identify two papers that included patients who underwent hemicraniectomy for related pathology after a different vaccine, so we believe our searches were adequate.

The Ad26.COV2.S vaccine has been under scrutiny and close monitoring since multiple cases of CVST were associated with the vaccine. This scrutiny began during the phase III trial of the vaccine, which reported a single case of CVST [9]. However, the case was attributed to the patient’s pre-existing risk factors, and the study was continued. Following the vaccine's emergency use authorization in February of 2021, additional cases were reported, and its public use was paused by a joint decision from the FDA and CDC [22]. Their subsequent review determined that the benefit of the vaccine's protection against COVID-19 outweighed the risk of developing CVST following vaccination [23,24], and they lifted the recommended pause [25].

There have been many published datasets that further support this risk/benefit analysis by showing that the risk of developing CVST during a COVID-19 infection is much higher than developing CVST following the Ad26.COV2.S vaccine [18,26-30]. As vaccine boosters are approved and widely administered, it is important to keep this rare side effect in mind when evaluating patients in the weeks after their vaccine and to consider neurosurgical decompression for life-threatening IPH related to VITT-induced CVST.

## Conclusions

This case is the first of its kind to be published, and it demonstrates that neurosurgical decompression can be considered for IPH secondary to CVST associated with VITT from the Ad26.COV2.S vaccine. We encourage this case to be discussed when the guidelines for the treatment of CVST related to VITT are updated, and we encourage readers that may have had experience with neurosurgical decompression for this pathology to publish their cases and add to the limited literature available.
